# The health system and women experiencing violence: the dedicated pathway in Emergency Departments in Italy and the experience in the city of Rome

**DOI:** 10.3389/fsoc.2026.1681402

**Published:** 2026-04-13

**Authors:** Francesca Proia, Alice Marta Mauri

**Affiliations:** 1 ISSIRFA CNR (Institute for the Study of Regionalism, Federalism and Self-Government of the National Research Council of Italy), Rome, Italy; 2 IRPPS CNR (Institute for Research on Population and social Policies of the National Research Council of Italy), Rome, Italy

**Keywords:** anti-violence centers, Emergency Department, health system, health-sector integration, IPV, Rome, violence against women, women experiencing violence

## Abstract

Violence against women (VAW) is a global public health problem and the health sector has an especially important role to play, considering the serious health risks faced by women. Women who experience violence are, more than others, at risk of acute and chronic health problems, as well as premature death. Medical services—especially Emergency Departments (EDs)—play a crucial role in a system that aims to intercept VAW at an early stage and guarantee women’s fundamental rights. In 2017, Italy approved *National Guidelines for healthcare and Hospital Organizations on emergency and socio-healthcare assistance for women experiencing violence*. World Health Organization (WHO) and national policy guidelines state that healthcare providers must develop integrated and multidimensional responses to intervene in cases of violence. The study aims to draw attention to the implementation of the guidelines because of two reasons. The first one is that no systematic assessment has yet been conducted to determine how effectively they are being applied. The second one is about how an integrated and multidimensional response can be developed in a complex setting such as an EDs to support women experiencing violence. The ongoing research makes use of desk and field research. Regarding the desk analysis, the study used technical reports and scientific literature to reconstruct the origins, characteristics and goals of national guidelines. The field research was conducted in Rome, because of its historical significance as one of the longest-running experiences of this kind in Italy. The case was studied by analyzing documentation and conducting field interviews with three anti-violence association referees present in the EDs and by visiting their EDs workplaces. This contribution presents selected findings. Specifically, it: outlines the objectives of the National Guidelines; reconstructs the heterogeneity of organizations operating in the city of Rome; examines three experiences of healthcare services that host anti-violence practitioners; highlights critical issues in the implementation of integrated and sustainable measures; emphasizes the need for stronger engagement by regional and hospital authorities to ensure that the Guidelines can fulfill their potential within broader policies to combat male violence against women.

## 1 Introduction

The World Health Organization (WHO) has highlighted that violence against women is a major global health issue and a priority for public health (WHO, 1996, 1997, 2024): nearly one in three women has experienced domestic or sexual assault by a stranger, or both forms of violence, at least once in her life (WHO, 2021).

The physical and psychological health consequences are numerous (e.g., Miller and McCaw, 2019; Karakurt et al., 2021; Guo et al., 2023; Mehr et al., 2023; Calvillo et al., 2024; Uvelli et al., 2024) and not always immediately attributable to the violence experienced (WHO, 2013; Campbell, 2002; Mellano et al., 2024). Violence constitutes both an etiological and risk factor for physical and psychological disorders, which occur more frequently in those who are, or have been, in violent situations. For example, Intimate Partner Violence is among the leading causes of morbidity and mortality for women of reproductive age (Berkley et al., 1993; Webster, 2016), and thus a cause of premature death (WHO, 2013). It is also recognized as a risk factor in the calculation of Disability-Adjusted Life Years (DALYs) within the Global Burden of Disease.

Protecting the health of women experiencing violence means assigning a fundamental role to the healthcare system, which women tend to use more frequently (e.g., WHO, 2002; Hegarty et al., 2020; Tarzia et al., 2020; Villacis Alvarez et al., 2025; Song et al., 2025), in the context of policies to combat male violence against women (WHO, 2016; Re et al., 2019). Measures to combat violence against women must be planned in relation to the various domains capable of intercepting the phenomenon (Walby et al., 2014), including the public health sector. Therefore, it is essential that the various professionals within the system are able to recognize the phenomenon and provide a multisectoral response (WHA, 2014). This is also true because women are willing to talk about their situation if they meet healthcare personnel who inspire trust, act without judgment/exhibit non-judgmental behavior, and respect their decisions (Tarzia et al., 2020).

This paper outlines the objectives of the Italian *National Guidelines for healthcare and Hospital Organizations on emergency and socio-healthcare assistance for women experiencing violence* (DPCM 24/11/2017, 2018), which were adopted specifically to support healthcare personnel in their roles. It also presents the initial findings of an ongoing study on the implementation of these National Guidelines in the Latium Region, with a specific focus on Rome. A particular area of interest is how Emergency Departments (EDs) are organized in their care of women experiencing violence, and how organizational methods affect working practices. The objective is to understand how an integrated and multidimensional response can be developed in a complex setting such as an ED to support women experiencing violence, according to international (in particular WHO’s guidelines) and national policy guidelines.

The guidelines highlight the need to provide multidimensional responses through the integration of services; however, they do not specify how such integration should be operationalized. This lack of guidance makes it particularly relevant to investigate how different services can be integrated in the care of women experiencing violence within EDs. In existing literature, there is no single definition of integrated services. The concept is generally used to refer to three levels: health services with staff able to provide a multidimensional response directly; health services that offer multidimensional responses through collaboration between multiple professionals engaged as needed; health services that only provide medical services and delegate other needs to external local actors (Colombini et al., 2008).

Based on this classification, the study investigated the second model—where skills are structurally integrated—since this approach is widespread in Rome and began to take shape well before the national guidelines. The other models of integration in Roman EDs will be investigated in later stages of the research.

Finally, the study also aims to draw attention to the implementation of the guidelines, as no systematic assessment has yet been conducted to determine how effectively they are being applied. Current data only indicate the number of EDs that have formally adopted the Guidelines, and there remains a scarcity of studies examining their implementation. Addressing this gap is essential for understanding both the effectiveness of the measure and the critical issues that must be resolved.

The article therefore aims to (1) illustrate the objectives of the National Guidelines and (2) report on how these guidelines have been implemented in the city of Rome. The paper is organized into four sections: the first reports on the methodological choices adopted; the second illustrates the objectives of the guidelines; the third reports the initial results of the ongoing research, in particular an initial mapping of the different organizational models of hospitals in the city of Rome and some issues that characterize the experiences of anti-violence staff in the hospitals analyzed; finally, the last section highlights some issues that have emerged as critical.

## 2 Methodology

The ongoing research aims to map out how the Italian National Guidelines are implemented in the territories. To this end, a desk analysis was first conducted followed by a field study.

Regarding the desk analysis, the study made use of technical reports and scientific literature to reconstruct the origins, characteristics and goals of Italian National Guidelines. In particular, the study made use of policy documents and reports, as well as scientific literature. Policy documents include materials produced by international organism, like WHO, World Health Assembly (WHA), and by Italian organisms, such as the Ministry of Health, the Higher Institute of Health and specific technical committees like the Women’s Health Committee.^1^ These documents help to understand what the policies addressed are. The scientific literature, both Italian and international, focuses on public health and on the health impacts of male violence against women.

The field research was conducted in Rome, based on its historical significance as one of the longest-running experiences of this kind in Italy. The case was studied by analyzing documentation and conducting field interviews.

According to the National Guidelines, Italian Regions are required to promote the adoption of national guidelines within their territories: for this reason administrative documents adopted by the Latium region were analyzed.^2^ During this phase of the investigation, organizational documents relating to the National Guidelines were analyzed, using documentation available on hospital websites. Documents published on the websites of anti-violence associations—whose staff are also present in hospitals—were then analyzed to reconstruct the characteristics of the associations and their experiences in hospitals.

The field research was carried out in May and June 2025 by conducting tandem semi-structured interviews with three anti-violence association referees present in the EDs and by visiting their EDs workplaces. These interviews were conducted by the authors of this paper, who alternated between the roles of interviewer and co-interviewer, and involved a total of seven people: three coordinators (with both service coordination and women’s support roles) and four workers (with women’s support roles) from anti-violence centers located in emergency rooms. As is standard practice in Italian anti-violence centers, all representatives and operators are women with specific training on the subject of male violence against women, with different disciplinary backgrounds. The interviews investigated both the characteristics of the associations and their experience within EDs; on the latter, the focus was on establishment and development over time, operating methods, interaction with healthcare personnel, and dedicated spaces. The main objective was to understand to what extent their experiences complied with the guidelines. To this end, a thematic analysis was conducted on the transcribed interview texts.

## 3 EDs pathways for women experiencing violence: the main objectives of the Italian national guidelines

The WHO has emphasized the central role of hospital facilities and the need for integrated approaches (WHO, 2002, 2013, 2014), issuing guidelines to help recognize the effects of violence and to provide intervention strategies for women-centered care.

In several countries around the world, guidelines to support healthcare professionals have been implemented, albeit slowly and not without difficulty (García-Moreno et al., 2015). In Italy, the key role of hospital facilities has been recognized since 2008 (Commissione Salute delle Donne, 2008), leading to the development of several significant pilot projects across the country. However, it was Law no. 208/2015 that formally assigned a key role to the healthcare system and promoted the adoption of guidelines targeting all healthcare companies and hospitals with EDs, to activate or standardize the “Pathway for Women Experiencing Violence” (Prime Ministerial Decree of November 24, 2017).

The Guidelines specify what actions must be taken in EDs: during the triage phase, it is necessary to recognize the woman’s possible exposure to violence, even when not explicitly stated, and assign an urgency code to activate the Pathway and ensure timely medical evaluation.

This assessment must take place in a separate area that ensures protection, safety and confidentiality. This area, which is restricted from any other person, serves as a space for initial reception and information on specialized services. Here, the medical examination is performed, along with clinical and instrumental assessments, the collection of any forensic evidence useful for possible reporting by the woman or healthcare personnel, and a risk assessment using the Brief Risk Assessment for the Emergency Department—DA5.^3^

If the risk is medium/high, the activation of local specialized services is recommended. When immediate availability of these services is lacking, Short-Term Intensive Observation or temporary hospital stay (up to 36–72 h) may be used to guarantee protection and safety.

The discharge report must include the primary and secondary diagnosis codes related to violence, in accordance with the International Classification of Diseases, 9th Revision, Clinical Modification (ICD-9-CM).^4^

Finally, to manage the interaction, it is recommended to use clear and understandable language—even for women with sensory, cognitive, or relational disabilities—and a non-judgmental approach that fosters a relationship based on trust.

The competencies required to implement such Pathways must be ensured by the Healthcare Organizations through appropriate training for staff. These Pathways must also be integrated and promoted at a regional level. As of today, 62% of EDs in Italy have an implementation protocol for the National Guidelines (Italian Ministry of Health, 2023).

## 4 A path of many developments: implementation of guidelines in Rome’s emergency department

The guidelines thus represent an important innovation on a national scale, since healthcare systems intercept women in situations of violence and can help to estimate the extent and damage caused by the phenomenon. A central theme of the guidelines is that healthcare services should provide integrated and multidimensional responses. This raises the question of how EDs decide to integrate internal skills, by strengthening the skills of healthcare personnel or by integrating them into anti-violence helpdesk structures, thus making anti-violence operators available to intervene in cases. The experience of the municipality of Rome illustrates the heterogeneity of possible operational solutions.

The following sections present the initial results of the ongoing research. In particular: section 3.1 presents an initial mapping of the different organizational models based on an analysis of regional documents and hospital reports available online; section 3.2 presents the initial results of the analysis of the experiences of anti-violence centers that also have staff working in hospitals, this section draws on materials available online from associations and information gathered through interviews with operators.

### 4.1 Fieldwork results: document analysis about Rome EDs pathways for women experiencing violence

This section presents data emerging from the document analysis referring to Latium Region administrative acts and to the documents available on the websites of Rome’s public hospitals. This section presents an initial mapping of the different organizational structures adopted in the EDs.

The Latium Region adopted the National Guidelines in 2019 and urged the 48 regional HO with EDs to adopt the Guidelines and implement the Pathway. At the same time, it recommended the gradual adaptation of pre-existing initiatives and required the appointment of a liaison officer for the regional authority and inter-institutional anti-violence network, to monitor the implementation of the regional Pathway.

To date, there are 22 EDs in Rome, including the largest hospital in Europe, Policlinico Umberto I, whose ED handles approximately 140,000 admissions per year. A desk analysis of the institutional websites of the HOs revealed that just 9 EDs (approximately 41–42%, far below the National outcome) have activated Pathways for women experiencing violence (Table 1).^5^

**Table 1:** Hospital by type of emergency department, organizational status and local health authority (Asl).

Hospital	Type of ED	Organizational status/Asl
G. Battista Grassi	General	Public/Asl Roma 3
Madre G. Vannini	General	Accredited private
San Camillo-Forlanini	General, Pediatric and Obstetric	Public/Asl Roma 3
San Filippo Neri	General	Public/Asl Roma 1
Sant’Andrea	General	Public/Asl Roma 1
Sant’Eugenio	General	Public/Asl Roma 2
Policlinico Universitario A. Gemelli	General	Accredited private
Policlinico Universitario Tor Vergata	General, Dental	Public/Asl Roma 2
Policlinico Universitario Umberto I	General, Ophthalmic, Pediatric, obstetric, Hematological and Dental	Public/Asl Roma 1

The implementation of pathways for women experiencing violence can be deployed in many ways. These pathways are based either on strengthening the skills of healthcare personnel and/or on integrating anti-violence center staff within the hospital facilities. Namely, in Vannini Hospital the Pathway is managed exclusively by healthcare staff; in two different cases, there is collaboration between healthcare staff and external organizations, including on-demand activation (e.g., Sant’Andrea Hospital with the Italian Red Cross; Sant’Eugenio Hospital with the Be Free cooperative). The standard implementation consists in an anti-violence center (or help desk) staffed with specialized personnel at HO premises.

A unique case is the activation of the “Percorso Aiuto Donna” (Women’s Help Pathway), which involves an entirely local health authority, ASL Roma 2. This program allows any woman identified at any ASL facility, not just EDs, to begin a psycho-social support Pathway. The woman is taken over by a team composed of psychologists, social workers, and midwives (supported by a pediatrician in the case of minors who are direct or indirect victims of violence). Throughout the entire process, the woman is followed by a Case Manager, who is either a midwife or a nurse (Asl Roma 2—Distretto 7, 2023).

Based on this preliminary survey, our research has focused on Emergency Departments that host anti-violence units managed by local feminist organizations, which also operate other territorial anti-violence centers and/or women’s shelters. Two of these Pathways were created before the issuance of the National Guidelines, and one was established afterward (Table 2).

**Table 2:** Anti-violence centers in ED by year of activation, hospital, operator, operator in other ED and anti-violence centers in Rome.

Anti-violence center	Year of activation	Hospital	Operator	Operator in other Ed	Operator and other AVC
Codice rosa*	2008	Policlinico Universitario Umberto I	Differenza donna	3	6**
Sportellodonnah24	2009	Ospedale San Camillo-Forlanini	Be Free	1	4***
S.O.S. Lei	2023	Policlinico Universitario A. Gemelli	Assolei	0	4

* It should be noted that the name Codice Rosa refers to all services provided by Differenza Donna at emergency departments as part of the Emergenza Codice Rosa project, which is also implemented at other hospitals that were not considered in this phase of the research.

** Differenza Donna also manages 4 anti-violence centers within the Latium Regional Health Authority and also in Latium and is active in the regions of Campania and Basilicata too.

*** Be Free manages another 3 anti-violence centers in Latium and is active in the regions of Abruzzi, Molise ed Umbria.

### 4.2 Fieldwork results: interviews: key elements of the three anti-violence centers operating within EDs

The three case studies show different organizational models, both in terms of the methodological approaches of the associations, all of which are feminist in origin, and in the specific features of the healthcare organizations. However, they all face a series of common daily challenges: ensuring support continuity and sustainability, securing dedicated spaces, training for both operators and healthcare staff, coordinating with territorial services, and managing high-risk situations.

The key elements presented in this section emerged during interviews with coordinators and operators, from on-site visits and from the analysis of reports produced by associations and/or information presented at public meetings or on their official websites.^6^

#### 4.2.1 Activation

Before the National Guidelines, two Pathways were created on the initiative of associations and with public funding. In 2008, the association “Differenza Donna” participated in a call for proposals from the Department for Equal Opportunities of the Italian Presidency of the Council of Ministers and launched “Emergenza Codice Rosa” a collaboration with the Social Services and Emergency Department staff at Policlinico Umberto I. In 2009, the association “Be Free” received funding from the Latium Regional Council for a context and needs assessment aimed at activating the service “Sportellodonnah24.” In the case of Policlinico Universitario A. Gemelli case, the center “SOS Lei” started in 2023 by the initiative of the healthcare organization, following a training program for medical and nursing staff provided by the association “Assolei,” which was selected as anti-violence center for the emergency department.

#### 4.2.2 Continuity and sustainability

“Emergenza Codice Rosa,” established in 2008 as a telephone help desk, obtained a dedicated space within the Policlinico Umberto I hospital in 2012 through new public funding. However, once the project expired, the initiative continued solely through self-funding until it was forced to shut down due to the COVID-19 emergency. During this period, it continued to guarantee its support through other local anti-violence centers also managed by the association.

“*We moved our number to one of our anti-violence centers, where it continued to answer the calls that came in. And we tried to ensure phone availability or, in any case, the possibility of welcoming women in our other locations.*” [interview]

It only reopened in May 2024, when a one-year agreement was signed between the HO and Differenza Donna, supported by Latium Region as part of a memorandum of understanding with the Latium Regional Prosecutor’s Office and the Lazio Psychologists’ society. The failure to renew this agreement led to the closure of the help desk on May 31, 2025. As of July 30, 2025, it has not been possible to obtain any information regarding any new Convention.

“Sportellodonnah24,” after a preliminary phase, obtained a direct agreement in 2009 with the San Camillo-Forlanini Hospital, lasting 2 years. When the agreement was not renewed, the gap was bridged by the voluntary work of the operators, who “continued to staff the help desk out of a sense of responsibility and respect for the women being supported” [interview]. Between 2012 and 2014, due to insufficient State funding, the cooperative initiated self-financing initiatives and partnerships to supplement available resources. Since 2018, “the service has become stable, directly integrated with the hospital’s health policy,” and “every two years, the hospital issues a public tender for the service through public procurement platforms” [interview]. During the COVID-19 emergency Sportellodonnah24 alternated between in-person and on-call phone support, struggling with the difficulty of organizing interviews in a hospital already under extreme pressure due to the pandemic and related restrictions.

“*We were on call from March 2020 until May, then they let us return only for the summer, and in October they put us back on call. We were back in person only in June 2022.*” [interview]

“SOS Lei” was created in 2023 thanks to a two-year agreement, later renewed, between the Policlinico Universitario A. Gemelli and Assolei, with no financial burden on the HO. The only resources available to the help desk, to partially reimburse the expenses of the operators providing reception and on-call support, come from a private company (Wind 3) following an agreement with the hospital. An internal fundraising campaign accomplished by Wind 3 raised the necessary amount to extend the opening time.

#### 4.2.3 Organizational models

From an organizational standpoint, the most relevant aspects are opening hours, number of staff members and their shift rotation, methodology used during interviews with women, and support pathways management.

All three Pathways ensure constant availability (on-call) but differ significantly in their weekly schedules as follows: Codice Rosa: Monday to Friday, 9:00 a.m. to 5:00 p.m.; SOS Lei: 9 h per week, spread across 3 days and Sportellodonnah24: 24/7, 365 days a year.

Codice Rosa and SOS Lei ensure phone availability and, if necessary, in-person presence beyond the standard schedule.

“*We were able to experience firsthand the difference between being physically present and just being on the phone. […] Talking on the phone with a stranger is complicated: we are already strangers, and the women who come here are usually seeking medical care, not an anti-violence center. There's that moment when we have to help the woman understand why it might be useful to talk with us, not just get the X-ray… On the phone, we often scheduled follow-up appointments in the following days [when we couldn’t come in person), but many women did not show up. When we meet them right there (upon access to the Emergency Room], they have our contact info, and know we are here 24/7—that is what brings them back later, when they are ready.*” [interview]

With reference to the number of operators and shift organization, the following patterns should be considered: Codice Rosa has 4 dedicated operators (including the coordinator), who cover daytime shifts and on-call availability. Two operators are present for each shift. SOS Lei has 12 operators rotating across all their anti-violence centers, including the one in the hospital. Among them, one operator is designated (on rotation) for phone availability. This operator ensures coordination between the healthcare organization and any additional resources needed in a specific circumstance they might face at ER level. Two operators are present per shift. Sportellodonnah24 has 7 dedicated operators. Each of them covers a total of 24 h per week (two 6-h daytime shifts and one 12-h night shift). For sustainability reasons, only one operator is present per shift, unlike in territorial anti-violence centers, where interviews are always conducted in pairs.

The issue of shift rotation and co-presence is closely linked to how the first access interview is handled at emergency department level and then returned for follow-up sessions. In the case of Sportellodonnah24, interviews necessarily take place with one operator, unlike the territorial anti-violence centers. Codice Rosa and SOS Lei both have two operators present, but with different approaches. In Codice Rosa, operators change between sessions, which is standard practice for that association’s anti-violence centers. In SOS Lei, the same operators follow the case throughout.

“*Our method is to always conduct interviews in pairs, never alone, because doing it alone gives the impression of a psychotherapy session. Each of us has specific skills, but here we are anti-violence center operators, specifically trained. That allows us to create a shared space with a woman, a space for exchange and openness. It is incredibly powerful, because it shows women that nobody here is trying to teach them anything.*” [interview]

“*We try to keep the same people involved to avoid creating a sort of secondary victimization—meaning, making women repeat their story again and again […] A woman builds trust with the operator, so we try to avoid bouncing her from one staff member to another. But that’s our style, I must say—it’s not the case in all anti-violence centers.*” [interview]

The personalized Pathway for escaping violence is promoted differently by each of the three services: Codice Rosa always follows women on-site, just like territorial anti-violence centers. SOS Lei agrees with the woman whether to continue on-site or at another territorial anti-violence center. Sportellodonnah24 prefers to refer women to other territorial anti-violence centers.

“*We can't take charge of them here, firstly because it's hard to get them back to the ER if they don’t have an open medical file, and secondly, we’ve seen that, emotionally, it’s not helpful for them to return to the emergency room every time for an interview and be reminded of why they came in the first place. So, we tend to refer them to territorial anti-violence centers depending on where they live, or whichever is more convenient, and makes them feel safer.*” [interview]

#### 4.2.4 Dedicated spaces

Codice Rosa at Umberto I Hospital started as a telephone help desk, partly due to the difficulty of finding an appropriate space within the hospital which was only later identified in a room within the Department of Gynecology and Obstetrics but located far from the main Emergency Department. This space is used for interviews with women who have already been welcomed and have started a pathway with the anti-violence center. The first contact takes place in an ED room, in the presence of a multidisciplinary team.

“*We try to carry out this first interview in a protected environment: there is a dedicated room where—if the woman is able to walk independently and has no health limitations—we go and begin the interview.*” [interview]

SOS Lei is located within the Oncology Center of Policlinico Gemelli, in a protected room, though also separate from the ED.

Sportellodonnah24, on the other hand, has found a stable location within the Emergency Department itself.

“*In a room with two doors: one opens into the Emergency Room waiting area, and the other into the inner Triage area. […] This location offers discretion and protection to women, who are often accompanied to the ER by the abusive partner and therefore unable to take initiative while with him in the waiting area. Once they enter Triage, however, they can access the Service.*” [interview]

#### 4.2.5 Training

Beyond the initial and ongoing training common to all staff at territorial anti-violence centers, the operator training program includes tailored instruction with specific modules and a shadowing period within the specific center.

“*Our training program includes lectures and shadowing periods, whether it’s at a territorial anti-violence center, a shelter, or an anti-violence center within a hospital. It’s clear that if you’ve done your training at a territorial anti-violence center, you can’t automatically come work here. But if you have trained here, then you can go to a territorial anti-violence center. There are relevant differences here, even just considering the entire medical-legal aspects.*” [interview]

Healthcare personnel training is also managed by the associations, which consider it an essential experience for exchanging knowledge and sharing skills. Training should not be a one-time event but rather a continuous learning process. Namely, the high turnover of doctors and nurses and the bureaucratic and organizational complexity are the most relevant challenges the healthcare staff face when attending training sessions.

“*Healthcare workers operate under emergency conditions, in the chaos of the emergency room… They deal with bureaucracy, delays, internal stress from managing all the situations. It’s not always possible to do everything; even just coming to our training sessions required authorization, time, opportunity. It wasn’t easy, because they just don’t have the time, they’re always working in emergencies!*” [interview]

Daily operational collaboration between professionals with different expertise and perspectives is also highly valued, creating a mutual learning process on the ground.

“*When you talk to the women, you also try to convey information to the healthcare staff: ‘I’m saying this to the woman, try to listen too, because this is the change you need to make if you have thought that women experiencing violence are fragile, have issues with their families, suffer from emotional dependency, or are caught in conflict.’ In that dialogue with the woman, I demonstrate that everything you have been taught in your training, I change it. Because in that two-way exchange between operator and woman, you see that violence is a spiral that drags you down, annihilates you, wipes you out, strips you of power, even materially.*” [interview]

“*You do training on the ground, and it is an ongoing process. Every case that comes in becomes a reason for further learning, and then the next day you have follow-up: ‘How did it end with that woman? What did you do?’ Unfortunately, or not, that is how you learn in a hospital: not in classroom, but on the ground.*” [interview]

#### 4.2.6 Networking

The anti-violence centers within hospitals actively build operational networks with the HO and local services, both specialized and general.

“*Without the network, we can’t make it… for us, the network is crucial: the network of associations, the formal network of mental health centers, law enforcement agencies, social services, etc. We keep networking every day. […] Every anti-violence center builds and maintains its own network of contacts in its local area (and even beyond), and these are then compiled by our organization, where we can centralize the network. We centralize all our connections, making them accessible to all our colleagues working in different areas.*” [interview]

“*The cases that require social services are managed together with the hospital’s Social Services, who have an edge compared to reaching out to local social services… they are colleagues, they have direct phone numbers, whereas we always should go through more formal channels. Still, the activation of the network is entirely up to us!*” [interview]

At the same time, when healthcare organizations engage in formalizing these networks, they often fail to include all the key players necessary for the integrated care of women experiencing violence, and do not always involve the anti-violence centers operating within hospitals.

“*There have been various working groups [for drafting a network protocol], but I have to say we were somehow left out. They call the territorial anti-violence center because they need it, and formally it has to be part of the table. But the hospital-based anti-violence centers sometimes gets bypassed, because the territorial facility is already there. There is an assumption that we only deal with emergencies -which, to be fair, is true- but a relevant part of our work consists in activating the external support network.*” [interview]

“*The hospital mostly maintains network relations with law enforcement, not with anti-violence centers or shelters… And that is really a weakness since we do not have safe spaces where we can welcome women.*” [interview]

This inevitably affects the timing of a woman’s discharge, especially when specialized services cannot intervene due to limited availability of emergency shelters, and the Hospital is not able to activate Short-Term Intensive Observation, namely when children are involved.

“*The cases where a woman is discharged to go to a shelter are very few, since in general shelters in Rome don’t admit women in emergency conditions. So, we end up finding temporary solutions: either placing them with the Social Operations Room of Roma Capitale or within private social facilities… Or, alternatively, what we do – which is the result of long-standing battles! – Is keep her within the hospital and activate the Short-Term Intensive Observation.*” [interview]

## 5 Concluding remarks

The three analyzed pathways unveil how the adoption of the National Guidelines has not played a decisive role in their establishment. To date, the presence and functioning of the pathways still seem to be deeply linked to the problem-solving skills of anti-violence centers and their staff, their initiative and their constant activation.

Integration within the structures can be different, beyond the mere coexistence of complementary competencies. Constant proximity of both healthcare and specialized staff highlights the advantages of synergic intervention on the patient, showing how collaboration helps overcome siloed knowledge. Moreover, it enables the development of shared languages, approaches, and intervention methodologies thus achieving the guideline targets and reworking the assistance context for women experiencing violence. It also enables continuous training that would be impossible otherwise. Training and awareness-raising efforts should never be seen as an acquired result, but as something that must be maintained over time (Busi et al., 2023). This is essential due to the high turnover in emergency departments, the shortage of staff, and the need to keep technical skills up with evolving legal standards (e.g., Law no. 168/2023 introduced new crimes prosecutable ex officio, which both healthcare professionals and anti-violence staff must be informed about).

Considering this need for constant proximity, in our case studies it has been observed that two out of the three services are unable to guarantee such an arrangement, either in terms of physical presence or available space within the general ED. However, they report that working jointly during interviews with medical staff increases the potential for cross-contamination of knowledge and practices, which increases the ability of medical staff to identify the warning signs of violence. This contributes to a key objective of the Guidelines: to equip emergency department staff to recognize, name, and intervene in the damage caused by violence. Even in a challenging environment often seen as unfit for addressing psycho-social issues, and where various defensive mechanisms hinder the ability to act (Borsari et al., 2017).

In such a context, where healthcare personnel face complex situations (e.g., unpredictability, heavy workloads, limited staff, short time with patients, potential conflict with users, language and communication barriers), the presence of specialized anti-violence personnel is a valuable and necessary resource to ensure an adequate response.

Monitoring the implementation of the Pathway remains essential, as it ensures access to a fundamental right: women’s health protection. The monitoring should include a specific focus on data collection and analysis. This is essential not only for a deeper understanding of the phenomenon of violence and its consequences for the health of the women who experience it, but also for guiding effective policies, both to update existing projects and to create new pathways in healthcare facilities that do not yet have them—a number that is quite significant.

The findings of this initial phase of research have highlighted the potential and critical issues of this integration model. In the future, the study will need to examine other models in greater depth and then systematize all the information for an adequate review.

*Every emergency room must be able to respond adequately, even the most remote one. […] Codice Rosa is a litmus test for understanding the functionality of certain healthcare services, and it also reflects the level of cultural awareness present in a healthcare organization* (Commissione parlamentare di inchiesta sul femminicidio, 2024).

## Data Availability

The original contributions presented in the study are included in the article; further enquiries may be addressed to the corresponding author.

## Footnotes

1 At this stage of the study, no analysis of similar guidelines adopted in other countries has been conducted.

2 The LARA (Regional Laws and Acts Against violence; https://lara.viva.cnr.it/) database was used to analyze the Region’s documents (Proia, 2023).

3 DA5 is a standardized and validated tool which “can help predict which victims may be at increased risk for severe injury or potentially lethal assault and can aid clinicians in differentiating which patients require comprehensive safety interventions” (Snider et al., 2009). It consists of 5 items to be assessed during the interview with the woman: Has the physical violence increased in frequency or severity over the past 6 months? Has he ever used a weapon or threatened you with a weapon? Do you believe he can kill you? Have you ever been beaten by him while you were pregnant? Is he violently and constantly jealous of you? Answering “yes” to 3 of these questions indicates a high risk of lethal violence or serious abuse.

4 The ICD9-cm diagnosis codes are: 995.50 minor abuse/maltreatment; 995.53 minor sexual abuse; 995.80 adult abuse/maltreatment; 995.83 adult sexual abuse; 995.51 psychological violence against minors.

5 The information is based on content from the websites of different hospitals, in particular: https://www.aslroma2.it/; https://roma.figliedisancamillo.it/; https://www.aslroma1.it/presidi-ospedalieri/presidio-ospedaliero-san-filippo-neri; https://www.aslroma2.it/index.php/organizzazione/ospedali/ospedale-s-eugenio; https://www.aslroma3.it/ospedale-g-b-grassi/; https://www.ospedalesantandrea.it/; https://www.policlinicogemelli.it/; https://www.policlinicoumberto1.it/; https://www.ptvonline.it/; https://www.scamilloforlanini.rm.it/.

6 Further information is available on the associations’ websites: https://www.assolei.it/; https://www.befreecooperativa.org/; https://www.differenzadonna.org/.
